# Collybistin and gephyrin are novel components of the eukaryotic translation initiation factor 3 complex

**DOI:** 10.1186/1756-0500-3-242

**Published:** 2010-09-21

**Authors:** Andrea L Sertie, Gustavo de Alencastro, Vanessa J De Paula, Maria Rita Passos-Bueno

**Affiliations:** 1 Department of Genetics and Evolutive Biology, Institute of Bioscience, University of Sao Paulo, Brazil; 2 Laboratory of Neuroscience LIM-27, Department and Institute of Psychiatry, Faculty of Medicine, University of Sao Paulo, Brazil

## Abstract

**Background:**

Collybistin (CB), a neuron-specific guanine nucleotide exchange factor, has been implicated in targeting gephyrin-GABA_A _receptors clusters to inhibitory postsynaptic sites. However, little is known about additional CB partners and functions.

**Findings:**

Here, we identified the p40 subunit of the eukaryotic translation initiation factor 3 (eIF3H) as a novel binding partner of CB, documenting the interaction in yeast, non-neuronal cell lines, and the brain. In addition, we demonstrated that gephyrin also interacts with eIF3H in non-neuronal cells and forms a complex with eIF3 in the brain.

**Conclusions:**

Together, our results suggest, for the first time, that CB and gephyrin associate with the translation initiation machinery, and lend further support to the previous evidence that gephyrin may act as a regulator of synaptic protein synthesis.

## Findings

Gaining deeper insights into the roles of the molecular players that mediate synapse formation and regulation is crucial for understanding brain functions and human disorders that affect learning and other cognitive skills, such as autism spectrum disorders. Despite our expanding knowledge in this area, the functions of several synaptic proteins, mainly those regulating the development and plasticity of inhibitory synapses, remain to be explored further. One such synaptic component is collybistin.

Collybistin (CB) is a brain-specific member of the family of guanine nucleotide exchange factor (GEF) proteins for small Rho-like GTPases [1]. Several CB splice variants have been identified in rodent brain and spinal cord [1,2]; all variants harbor the catalytic DH and membrane-binding PH tandem domains, but differ by the presence of an N-terminal SH3 domain, and by alternative C-termini, which may contain a α-helical coiled-coil motif. The human CB homologue (hPEM-2) was shown to catalyze specific nucleotide exchange on Cdc42 in fibroblasts and to activate actin polymerization and changes in cell morphology [3].

CB binds to gephyrin [1], a major postsynaptic scaffolding protein required for the clustering of both glycine and major classes of GABA_A _receptors [4-7]. Importantly, CB has been implicated in the translocation of gephyrin from large cytoplasmic aggregates to the plasma membrane of cultured cells, and the PH domain of CB was shown to be required for this activity [8], whereas the SH3 domain seems to negatively regulate this activity [1,2]. Interestingly, it has recently been shown that the SH3 domain of CB interacts with neuroligin 2, a postsynaptic cell adhesion protein [9], and with the GABA_A _receptor α2 subunit [10], and these interactions seem to relieve the inhibitory effect of this domain, thus rendering the CB_SH3+ _variants, the predominant brain and spinal cord isoforms, active in targeting gephyrin scaffolds to the plasma membrane.

Consistent with CB regulating recruitment of gephyrin scaffolds to developing inhibitory postsynaptic sites, CB-deficient mice show loss of postsynaptic gephyrin and GABA_A _receptors clusters in the hippocampus and the amygdala, which is accompanied by impaired GABAergic transmission, altered hippocampal synaptic plasticity and behavioral abnormalities in the mice [11-13]. The importance of this protein has been further demonstrated by the identification of mutations in human CB gene (*ARHGEF9*, mapped at Xq11.1) in patients with diverse neurological abnormalities, including hyperekplexia, epilepsy, mental retardation, insomnia, aggressive behavior and anxiety [2,14,15].

Aside from regulating gephyrin and GABA_A _receptors deposition at inhibitory synapses, little is known about what other functions CB might subserve. In this study, in an attempt to gain further insight into the role of CB in neuronal development and function, we sought to identify novel human CB-interacting partners. Our results demonstrate that CB is associated with the translation initiation complex, and suggest that CB, along with gephyrin, may be involved in the regulation of protein synthesis at postsynaptic sites.

## Materials and methods

### Yeast Two-hybrid screening

Yeast two-hybrid screening was conducted using Matchmaker GAL4 two-hybrid system 3 (Clontech, BD Biosciences). Reagents and amino acids required for making standard dropout (SD) plates for prototroph and colorimetric screening were obtained from Sigma-Aldrich.

#### Plasmid constructs

Full-length human CB cDNA (encoding amino acids 1 to 516) and a truncated form lacking the cDNA sequence for the N-terminal SH3 domain (encoding amino acids 64 to 516) was cloned downstream of the GAL4 DNA-binding domain in pGBKT7 vector (plasmids pGBKT7-CB and pGBKT7-CB_SH3-_, expressing the bait proteins GAL4BD-CB and GAL4BD-CB_SH3- _respectively). Full-length human gephyrin cDNA (enconding amino acids 1 to 769) was cloned downstream of the GAL4 activation domain vector pGADT7 (plasmid pGADT7-gephyrin, expressing the prey protein GAL4AD-gephyrin).

#### Testing the bait protein

After construction of the bait plasmids, the yeast strain AH109 was transformed and evaluated for bait proteins expression, transcription activation in the absence of a binding partner and effect on mating efficiency. These initial control studies suggested that human CB would be effective bait in the screen.

#### Yeast mating and screening

A human fetal brain cDNA library in the GAL4 activation domain vector pACT2 pretransformed into the yeast strain Y187 was screened in a yeast two-hybrid assay through large scale mating to AH109 expressing GAL4BD-CB following the manufacturer's instructions. Mating efficiency was determined to be within the acceptable limits according to the manufacturer's protocol. Diploid cells were screened for growth on SD agar plates lacking leucine, tryptophan, adenine, histidine (to verify the expression of *ADE2 *and *HIS3 *reporter genes) and containing X-α-Gal (to verify the expression of *LacZ *reporter gene) and 5 mM 3-amino-1,2,4 triazole (3-AT) for 7 days at 30°C. Positive colonies were subjected to a new phenotype test for segregation of independent clones, preventing the presence of two or more plasmids derived from the library in a single clone. Thus, positive colonies were streaked on SD/-Leu/-Trp/-Ade/-His/X-α-Gal/5 mM 3-AT plates and segregation was confirmed by the observation of white and blue colonies. Positive blue clones were further evaluated for beta-GAL expression by colony-lift filter assay, according to the manufacturer's instructions. Total plasmids were extracted from these putative positive interaction yeast colonies and transformed into *Escherichia coli *DH5α competent cells (Invitrogen) followed by isolation of the pACT2-cDNA plasmids. To retest the positive interaction, individual clones of pACT2-cDNA were cotransformed with pGBKT7-CB or pGBKT7-CB_SH3- _into AH109 and analysed aforementioned. The putative positives were also tested against plasmid pGBKT7 alone to discard false positives. After confirmation of interaction, pACT2-cDNAs were sequenced and analysed by comparison to GenBank sequences.

### Antibodies

Mouse monoclonal anti-Myc antibody was obtained from Clontech. Mouse monoclonal anti-Flag M2 antibody was obtained from Sigma. Mouse monoclonal anti-Collybistin antibody was obtained from BD Transduction Laboratories. Goat polyclonal anti-Gephyrin (R-20), anti-eIF3B (N-20), and anti-BMP1 (A-20) antibodies were purchased from Santa Cruz Biotechnology. Rabbit polyclonal anti-eIF3H and anti-Sox2 antibodies were purchased from Cell Signaling.

### Mammalian expression constructs

Full-length human CB and gephyrin cDNAs were generated by PCR and subcloned into a pcDNA3.1 vector (Invitrogen) that contained sequence encoding the Flag epitope tag at the 5' end of the polylinker to allow expression of N-terminally Flag-tagged proteins. Similarly, full-length human CB, gephyrin and eIF3H cDNAs were generated by PCR and subcloned into a pExchange-4 vector (Stratagene), which is an N-terminal c-Myc tagging vector that allows expression of N-terminally Myc-tagged proteins. All constructs were sequence verified.

### Cell culture and transfection

HEK 293T cells were grown in Dulbecco's modified Eagles medium supplemented with 10% fetal bovine serum (FBS) and 100 U/ml penicillin/streptomycin. The cultures were maintained at 37°C in a humidified atmosphere of 95% air and 5% CO2. All transfections were carried out using SuperFect (Qiagen) according to the manufacturer's instructions.

### Preparation of cell or mouse whole brain lysates

After 48 h of transfection, HEK 293T cells expressing different combinations of epitope-tagged forms of target proteins were washed with PBS and lysed in RIPA buffer (1 mL/100 mm plate) containing 50 mM Tris-HCl (pH 7.4), 150 mM NaCl, 1 mM EDTA, 1% NP-40, 0.1% SDS, 0.5% deoxycholic acid supplemented with protease inhibitors. Lysed cells were incubated in ice for 30 min, centrifuged at 14,000 rpm for 15 min at 4°C, and the supernatant was saved. Mouse brains were removed, frozen in liquid nitrogen, lyophilized, and homogenized in RIPA buffer (1 mL/200 mg). Brain lysates were kept on ice for 30 mim, and supernatants were collected after centrifugation at 14,000 rpm for 30 min at 4°C. Protein concentration was determined with Bio-Rad protein assay.

### Coimmunoprecipitation and immunoblot analysis

For immunoprecipitation from HEK 293T cell lysates, ~1.5 mg of protein were pre-cleared with protein G-Sepharose (GE Healthcare) and then incubated with antibody (4 mg) against the Flag epitope, overnight at 4°C on a rotating platform. For immunoprecipitation from mouse whole brain lysates, ~10 mg of proteins were pre-cleared with protein G-Sepharose and then incubated in the presence of antibodies (4 mg) against the eIF3H or eIF3B proteins, or irrelevant isotype-matched antibodies as negative controls, overnight with rotation at 4°C. Subsequently, all lysates were incubated with 50 mL protein G-Sepharose for 3-4 h at 4°C on a rotating platform. After centrifugation, beads were washed 3 times with RIPA buffer and once with 50 mM Tris-HCL pH 8.0. The bead-bound proteins were eluted with SDS sample buffer, resolved by SDS-PAGE, and analyzed by Western blotting. Expression of epitope-tagged recombinant proteins or endogenous proteins from whole-brain extracts was also analysed by loading equivalent amounts of lysates into the gel. For immunodetection, blots were incubated with the primary antibody solutions, followed by HRP-conjugated secondary antibodies. Bound HRP was detected using the enhanced chemiluminescence (ECL) system (Amersham Biosciences).

## Results

### Identification of eIF3H as a collybistin binding partner

**Figure 1:**
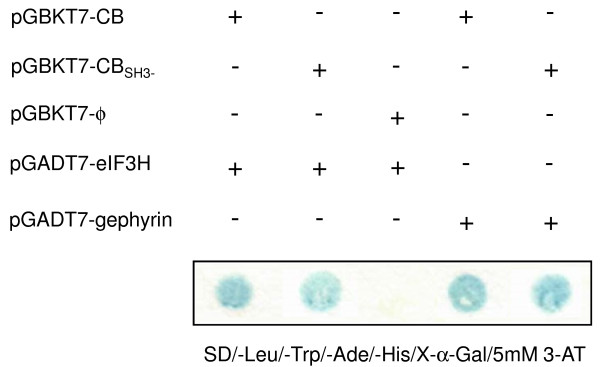
**Yeast two-hybrid assay**. Yeast strain AH109 cotransformed with pGBKT7-CB or pGBKT7-CB_SH3- _bait plasmids and pGADT7-eIF3H prey plasmid grew on SD/-Leu/-Trp/-Ade/-His/X-α-Gal/5 mM 3AT plates, whereas a control transformant, with a pGBKT7 empty vector and pGADT7-eIF3H, did not. As CB-bait specific positive interaction control, yeast strain AH109 was cotransformed with pGBKT7-CB or pGBKT7-CB_SH3- _and pGADT7-gephyrin vectors. Results shown are representative of at least three independent experiments.

To identify novel CB-interacting partners, we performed a yeast-two-hybrid screen of a human fetal brain cDNA library using the full-length human CB as bait. From ~2.7 × 10^6 ^clones screened, 8 positive clones were isolated using the most stringent reporter selection. To retest protein-protein interaction in yeast, library plasmids were rescued and cotransformed into the AH109 yeast strain with either the pGBKT7-CB plasmid (expressing the bait protein GAL4BD-CB) or an empty pGBKT7 vector (negative control). Four of these clones were judged to be true-positives and two of them contained the full-length coding sequence of p40 subunit of the eukaryotic translation initiation factor 3 (eIF3H). Yeast-two-hybrid assay using the human CB lacking the SH3 domain (bait protein GAL4BD-CB_SH3-_) showed that this truncated form of CB can also interact with eIF3H, indicating that this domain is not required to mediate the interaction with eIF3H (Figure 1).

### Collybistin and gephyrin associate with eIF3H in mammalian cells

To investigate whether CB and eIF3H associate in a mammalian cell context, coimmunoprecipitation assays were carried out.

**Figure 2:**
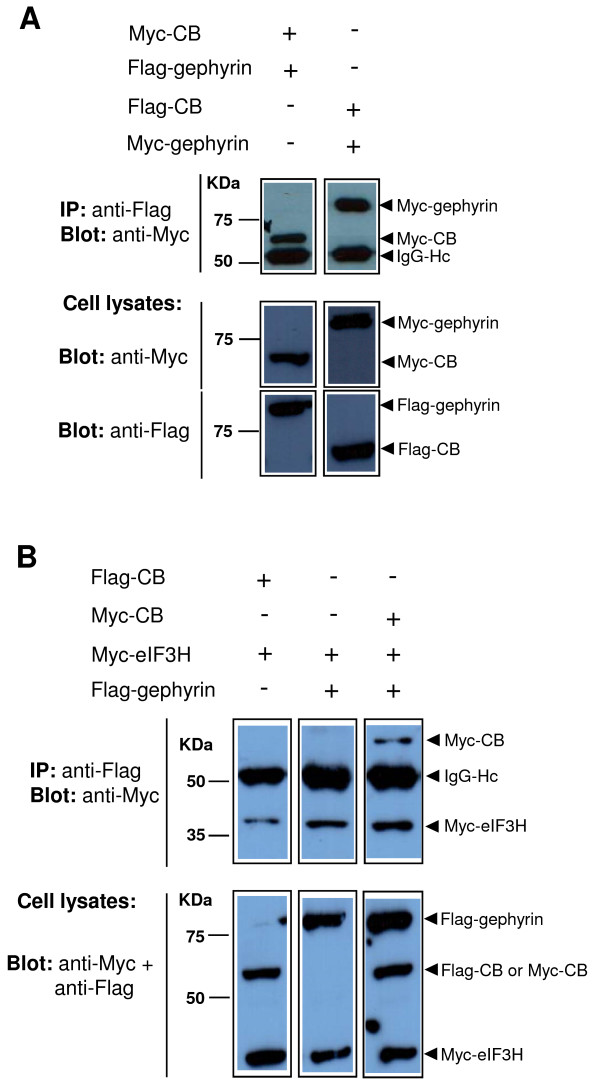
**CB, gephyrin and eIF3H interaction in a mammalian cell context**. 293T cells were cotransfected with plasmids enconding the indicated combinations of the epitope-tagged proteins: **(A) **Myc-CB (~62 kDa) and Flag-gephyrin (~95 kDa), Flag-CB (~62 kDa) and Myc-gephyrin (~95 kDa); **(B) **Flag-CB and Myc-eIF3H (~40 kDa), Myc-eIF3H and Flag-gephyrin, Myc-CB and Myc-eIF3H and Flag-gephyrin. As negative controls 293T cells were cotransfected with a plasmid encoding the Flag-tag alone along with plasmids encoding the Myc-tagged proteins (data not shown). Immunoprecipitations were carried out using anti-Flag antibody and immunoblots were performed using anti-Myc antibody (top panels). Coimmunoprecipitations of CB-gephyrin (**A**, top panels), CB-eIF3H (**B**, top left panel), gephyrin-eIF3H (**B**, top middle panel), CB-gephyrin-eIF3H (**B**, top right panel) were observed. In analyzing negative controls, no detectable protein coimmunoprecipitation was accomplished (data not shown). Expression levels of the ectopic expressed proteins were confirmed by direct immunoblotting of equivalent amounts of the lysates using anti-Flag and/or anti-Myc antibodies as shown in the lower panels. All results shown are representative of two-four independent experiments. IP = antibody used for immunoprecipitation; Blot = antibody used for immunoblot analysis; IgG-Hc = immunoglobulin heavy chain (~55 kDa).

Initially, HEK 293T cells were cotransfected with expression plasmids encoding Myc-tagged CB and Flag-tagged gephyrin or Flag-tagged CB and Myc-tagged gephyrin as positive control for protein interactions. Cell lysates were immunoprecipitated with anti-Flag antibody, followed by immunoblot analysis with anti-Myc antibody. We observed, as expected, that Myc-CB (Figure 2A, top left panel) and Myc-gephyrin (Figure 2A, top right panel) were present in the immunocomplex precipitated with anti-Flag antibody.

Next, HEK 293T cells were cotransfected with expression plasmids encoding Flag-CB and Myc-tagged eIF3H. Anti-Flag antibody was used to immunoprecipitate Flag-CB from cell lysates, and coimmunoprecipitated Myc-eIF3H was detected by immunoblotting with anti-Myc antibody (Figure 2B, top left panel). These results demonstrate that CB interacts with eIF3H in mammalian cells.

We then examined the possibility that gephyrin may also interact with eIF3H. Flag-gephyrin and Myc-eIF3H were transiently expressed in HEK 293T cells. Following immunoprecipitation with anti-Flag antibody, an immunoblot of this material with anti-Myc antibody showed coimmunoprecipitation of Flag-gephyrin and Myc-eIF3H (Figure 2B, top middle panel). These results demonstrate that gephyrin also interacts with eIF3H in mammalian cells. Whereas we cannot rule out the possibility that gephyrin may mediate the CB-eIF3H interaction in HEK 293T, which express endogenous gephyrin, CB seems not to be required for gephyrin-eIF3H association, since detectable levels of endogenous CB was not observed in HEK 293T cells (data not shown).

Finally, complex formation of CB, gephyrin and eIF3H was investigated. Myc-CB, Myc-eIF3H and Flag-gephyrin were transiently expressed in HEK 293T cells and the immunoprecipitation was done using the anti-Flag antibody. After immunoblotting with anti-Myc antibody, we observed that Myc-CB and Myc-eIF3H could be coimmunoprecipitated with Flag-gephyrin (Figure 2B, top right panel). Although we cannot rule out the possibility that these results are due to the formation of two independent protein complexes, CB-gephyrin and gephyrin-eIF3H, they suggest that these interactions are not mutually exclusive (as both could potentially form at the same time), and raise the possibility that CB, gephyrin and eIF3H can form a trimeric protein complex in mammalian cells.

### Collybistin and gephyrin associate with eIF3 in the brain

**Figure 3:**
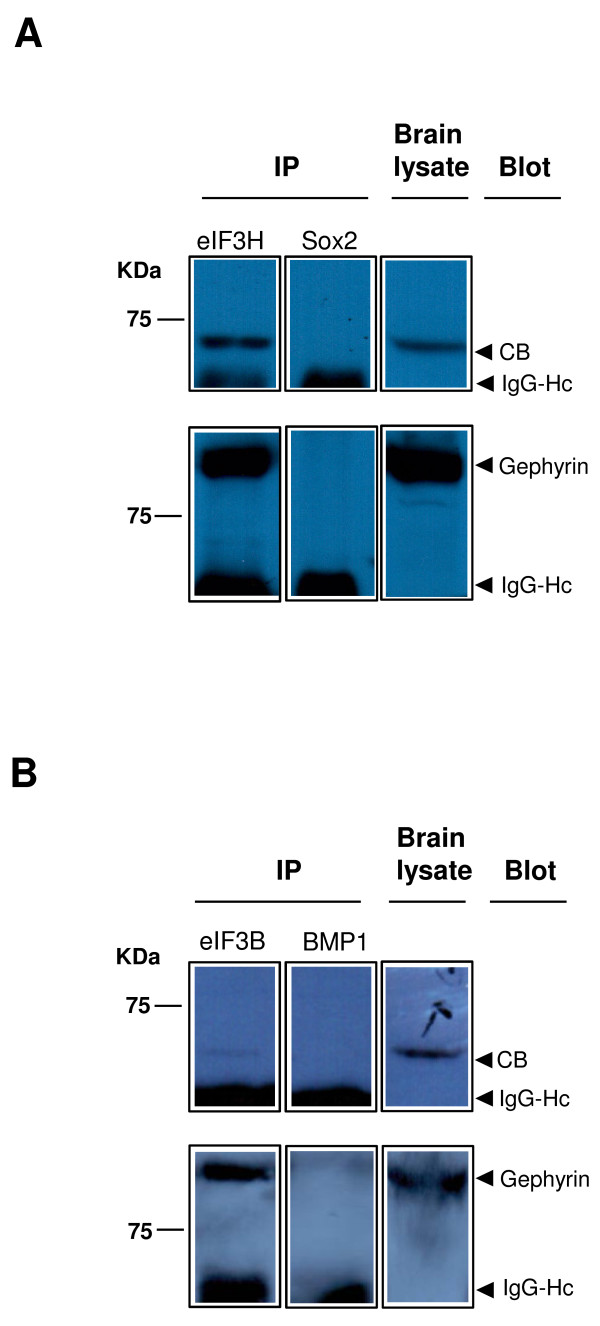
**Interaction of CB, gephyrin and eIF3 in the brain**. Adult mouse whole brain lysates were immunoprecipitated using **(A) **anti-eIF3H or **(B) **anti-eIF3B antibodies and endogenous CB (~62 kDa) and gephyrin (~95 kDa) were detected in immunoprecipitated samples using anti-CB and anti-gephyrin specific antibodies respectively. As negative controls, corresponding isotype-matched immunoglobulin G antibodies (including rabbit anti-Sox2 and goat anti-BMP1 antibodies) were used for immunoprecipitaion, and no coimmunoprecipitation of CB and gephyrin was observed. All results shown represent one of at least three independent experiments. Although a faint band corresponding to coimmunoprecipitated CB was detected using anti-eIF3B antibody for immunoprecipitation (**B**, top left panel), it is worth pointing that the same weak but specific CB band was observed in all the experiments, which suggest that CB also interacts with the eIF3 complex. IP = antibody used for immunoprecipitation; Blot = antibody used for immunoblot analysis; IgG-Hc = immunoglobulin heavy chain (~55 kDa).

We next examined whether CB and gephyrin associate with eIF3H in mouse brain. Immunoblot analysis of adult mouse whole brain lysates showed expression of endogenous CB, gephyrin and several subunits of the eIF3 complex, including eIF3H and eIF3B (also known as eIF3-p116) (data not shown). Since the antibodies available against CB and gephyrin were not suitable for immunoprecipitation, we used an eIF3H-specific antibody for immunoprecipitation followed by immunoblotting with anti-CB or anti-gephyrin antibodies. We observed co-immunoprecipitation of endogenous CB as well as gephyrin with eIF3H from mouse whole brain homogenates (Figure 3A, top left panel and bottom left panel respectively). No coimmunoprecipitation of collybistin and gephyrin were detected using an isotype-matched antibody (Figure 3A, top middle and bottom middle panels respectively). The same results were obtained when we used an antibody against eIF3B for immunoprecipitation (Figure 3B). These results indicate that CB and gephyrin associate with the eIF3 complex in the brain.

## Discussion

Here, we used the yeast two-hybrid (YTH) approach to search for proteins that associate with human CB and identified eIF3H as a potential interaction partner. Also, through YTH assay, we showed that the SH3 domain of CB is not required for interaction with eIF3H. Interaction of CB and eIF3H was further substantiated by coimmunoprecipitation of transiently expressed proteins in 293T cells. In addition, we showed that gephyrin also interacts with eIF3H in 293T cells and that CB, gephyrin and eIF3H seem to assemble into a trimeric protein complex. Finally, in mouse brain tissue, we could demonstrate the interaction of endogenous CB and gephyrin with the eIF3 complex. In conclusion, we showed by independent approaches that CB and gephyrin bind to eIF3.

Translation initiation in eukaryotes requires the participation of several eukaryotic initiation factors (eIFs), and the eIF3 is the largest and most complex of the eIFs, consisting of 13 subunits (designated eIF3A to eIF3 M in order of decreasing size) in mammalian cells [16,17]. eIF3 plays a central role in translation initiation and its regulation, including formation of the 43 S preinitiation complex (consisting of the eIF2-GTP-Met-tRNA ternary complex, eIF3, eIF1, eIF1A, eIF5 and the 40 S ribosomal subunit), binding of the 43 S preinitiation complex to the 5'-m^7^G-capped mRNA, and reaching the AUG codon [16,18]. In addition, recently eIF3 was also shown to provide focal points for translational control of gene expression in response to stimuli (such as nutrients, energy sufficiency, hormones and mitogenic agents) through association with mTOR and S6K1 [19,20], serine/threonine protein kinases which act as primary regulators of protein synthesis and cell growth. Thus, the eIF3 complex may act as the general scaffolding platform, bringing all components of the process into close proximity.

Although the roles played by CB and gephyrin in this scenario have yet to be defined, our molecular data on the physical interaction of CB and gephyrin with eIF3 suggest that these proteins may participate in translation initiation. It is particularly important to note that gephyrin was previously shown to associate with mTOR, and this interaction appears to be necessary for downstream signaling of mTOR [21]. In light of these findings, it is tempting to speculate that gephyrin acts, indeed, as a scaffold protein bridging together CB, eIF3 and mTOR, and that CB, maybe through its GEF activity, is the protein responsible for activation of mTOR signaling. One possibility is that CB may promote guanine nucleotide exchange on Rheb, a Ras-related GTPase that when linked to GTP activates mTOR's enzyme activity [22,23]. It is interesting to note that the *S. cerevisiae *protein Cdc24p, which shows both functional and sequence homology (26% identity) with human CB and also acts as GEF for Cdc42p, interacts with the yeast TOR protein complexes [24]. Alternatively, CB may have a direct effect on eIF3 assembly and/or function, independent on mTOR, or CB function may rely on translocation of the translation machinery to synaptic sites. We are currently performing follow-up experiments to address these important issues.

It has been well established that protein synthesis can occur in specific subcellular regions of a neuron in response to extracellular signal, and colocalization of the translational machinery together with cell surface receptors and signaling molecules near or at synapses may play important role in synaptic function and plasticity [25-28]. Importantly, mTOR pathways also have been implicated in local translation and synaptic plasticity in dendrite [29]. Results from our study thus provide initial support for the involvement of CB and gephyrin in the control of protein synthesis at inhibitory synapses, and also provide a framework for further investigation into the molecular mechanisms involved in local synaptic translation and in the pathophysiology of diseases associated with mutations in these proteins. Moreover, our results, together with results from previous studies, link CB and gephyrin to biological pathways associated with autism, which make these proteins also plausible candidates for autism susceptibility.

## Competing interests

The authors declare that they have no competing interests.

## Authors' contributions

ALS and MRPB conceived and designed the experiments. ALS, GA, VJDP performed the experiments. ALS wrote the paper. All authors read and approved the final manuscript.
